# Effect of Perineural Dexamethasone as an Adjuvant to Ropivacaine in Rectus Sheath Block for Radical Cystectomy: A Randomized Controlled Trial

**DOI:** 10.3390/jcm14155186

**Published:** 2025-07-22

**Authors:** Seung Hee Yoo, Min Hyouk Beak, Dong Hyeon Lee, Won-Joong Kim

**Affiliations:** 1 Department of Anesthesiology and Pain Medicine, College of Medicine, Ewha Womans University, Ewha Womans University Mokdong Hospital, Seoul 07985, Republic of Korea; yoosh0710@naver.com (S.H.Y.); minbeak@gmail.com (M.H.B.); 2 Department of Urology, College of Medicine, Ewha Womans University, Ewha Womans University Mokdong Hospital, Seoul 07985, Republic of Korea; leedohn@ewha.ac.kr

**Keywords:** radical cystectomy, rectus sheath block, dexamethasone, postoperative pain, analgesia, opioid consumption, rebound pain, ultrasound-guided regional anesthesia

## Abstract

**Background/Objectives**: Radical cystectomy performed via midline laparotomy is associated with substantial postoperative pain, frequently necessitating a high opioid consumption, which may impair immune function and delay recovery. The rectus sheath block (RSB) is widely used as part of multimodal analgesia to enhance postoperative pain control; however, the duration of analgesia is limited when using single-injection techniques. Dexamethasone has increasingly been used as a perineural adjuvant to prolong the effects of peripheral nerve blocks and enhance analgesia. This randomized controlled trial evaluated whether adding perineural dexamethasone to an RSB improves analgesic efficacy in patients undergoing a radical cystectomy. **Methods**: Fifty-two adult patients scheduled for radical cystectomy were randomly assigned to receive an ultrasound-guided bilateral RSB with either 0.25% ropivacaine alone or 0.25% ropivacaine combined with 4 mg dexamethasone per side after skin closure. Postoperative pain was assessed using a numeric rating scale (NRS) at 3, 6, 12, 18, 24, and 48 h following surgery. Cumulative intravenous patient-controlled analgesia (IV-PCA) in terms of fentanyl consumption and the incidence of rebound pain—defined as an increase in the NRS from ≤3 to ≥7 within 24 h after the block administration—were also recorded. **Results**: The dexamethasone group exhibited significantly reduced cumulative fentanyl consumption. Pain scores were consistently lower in the dexamethasone group compared with the ropivacaine-only group at all time points except 3 h postoperatively. The incidence of rebound pain was also substantially lower in the dexamethasone group. **Conclusions**: Perineural dexamethasone as an adjuvant to an RSB provides effective and prolonged analgesia, reduces opioid requirements, and lowers rebound pain incidence in patients undergoing a radical cystectomy.

## 1. Introduction

Midline laparotomy, commonly performed in colorectal, gynecological, upper gastrointestinal, and urological procedures, accounts for a substantial proportion of operations worldwide [1]. This approach, involving a vertical midline abdominal incision, is frequently associated with postoperative complications such as pain, pulmonary dysfunction, delayed gastrointestinal motility, and venous thromboembolism [2,3]. In radical cystectomy, which requires a large abdominal incision, intraoperative opioid requirements are typically higher, and patients often experience more-severe postoperative pain, which can negatively affect immune function [4]. In cancer surgery, maintaining perioperative immune competence is essential for reducing the risk of intraoperative tumor dissemination and postoperative cancer recurrence [5]. Moreover, the average age of patients undergoing a radical cystectomy continues to rise, with many presenting with comorbidities involving vital organs such as the heart and lungs [6]. Consequently, effective and balanced postoperative pain management in this population remains critical [7].

To optimize postoperative analgesia, multimodal strategies are commonly employed, combining non-opioid medications such as paracetamol and nonsteroidal anti-inflammatory drugs (NSAIDs), regional anesthetic techniques, and opioids. Single-injection peripheral nerve blocks (PNBs) are a key component, providing effective early postoperative analgesia [7]. PNBs have been shown to reduce opioid requirements, decrease postoperative nausea and vomiting (PONV), and facilitate earlier hospital discharge [8,9]. However, postoperative pain often persists for 24–72 h, and patients may experience a sudden increase in pain (“rebound pain”) as the nerve block wears off [10]. A retrospective cohort study of outpatients who received preoperative PNBs reported a 49.6% incidence of rebound pain. Identified risk factors included younger age, female sex, bone-related procedures, and the absence of perioperative intravenous dexamethasone [11].

Adjuvant drugs such as alpha-2 agonists, dexamethasone, midazolam, and NSAIDs can prolong local anesthetic duration and reduce dose-dependent adverse effects [12]. Among these, dexamethasone—a glucocorticoid commonly used to suppress inflammatory responses—has received considerable attention as an adjuvant in regional anesthesia. It is frequently co-administered with local anesthetics to extend the duration of analgesia provided by single-injection PNBs [13,14,15,16]. Dexamethasone has also demonstrated efficacy in reducing postoperative pain and opioid use across various surgical procedures. Its anti-inflammatory, immunomodulatory, and analgesia-prolonging properties make it a valuable component of multimodal analgesia strategies [17,18]. A single perioperative dose of dexamethasone has not been shown to increase the infection risk or delay wound healing; however, its use may be associated with transient hyperglycemia, particularly with repeated doses or in high-risk patients [19,20].

Thoracic epidural analgesia is considered to be the gold standard for postoperative pain control in abdominal surgery, but its use is often limited by contraindications and potential complications. Although serious adverse events are uncommon, risks include spinal epidural hematoma, abscess formation, and direct neural injury. Common side effects include hypotension, sedation, and pruritus, and its use is contraindicated in patients requiring anticoagulation—a scenario that is increasingly encountered in clinical practice [21]. Moreover, epidural analgesia can be technically challenging, with reported failure rates ranging from 6% to 37% [22].

As alternatives, the use of fascial plane block techniques targeting peripheral nerves of the abdominal wall has become increasingly widespread. A meta-analysis comparing continuous peripheral nerve catheters with epidural analgesia for laparotomy found no significant difference in pain scores during the first 48 h postoperatively [23]. Furthermore, a systematic review and meta-analysis of non-neuraxial analgesic techniques concluded that both the single-injection rectus sheath block (RSB) and transversus abdominis plane (TAP) block are effective in reducing pain and PONV within the first 6 h after surgery [24]. Continuous RSB and TAP blocks were also identified as being the most effective non-neuraxial analgesic techniques for reducing 24 h and 48 h morphine use, resting pain, and pooled opioid-related adverse effects [24].

The RSB is a regional anesthesia technique that is particularly suited for midline abdominal incisions. The abdominal wall is innervated by thoracic spinal nerves (T6–L1) that traverse the transversus abdominis and internal oblique muscles, forming a network within the TAP and near the deep inferior epigastric artery. The RSB targets these nerves as they pass from the posterior layer of the rectus sheath into the rectus abdominis muscle [25], providing effective analgesia across the central anterior abdominal wall, from the xiphoid process to the symphysis pubis [26,27].

To date, no studies have evaluated postoperative pain outcomes or the incidence of rebound pain after a single-injection RSB combined with perineural dexamethasone in patients undergoing a radical cystectomy. We hypothesized that adding dexamethasone to an RSB would reduce opioid use, improve postoperative analgesia, and lower the incidence of rebound pain. Therefore, this study aimed to compare postoperative pain outcomes and the incidence of rebound pain between patients receiving an RSB with and without dexamethasone.

## 2. Materials and Methods

This study was conducted in accordance with the Declaration of Helsinki and approved by the Institutional Review Board of the Ewha Womans University Mokdong Hospital (EUMC 2024-09-029-003, 25 November 2024). The study was registered with the Clinical Trial Registry of Korea (KCT0010081, cris.nih.go.kr, accessed on 26 December 2024.). The first patient was enrolled on 7 January 2025, and written informed consent was obtained from all participants.

### 2.1. Study Design and Participants

Patients scheduled to undergo a radical cystectomy for bladder cancer were eligible for inclusion. The inclusion criteria were age ≥ 20 years, American Society of Anesthesiologists physical status 1–3, and a planned elective radical cystectomy under general anesthesia with a single-injection RSB between January and May 2025. Exclusion criteria included coagulopathy, neuropathic disorders, allergy to local anesthetics, severe cardiopulmonary disease, systemic steroid use, uncontrolled diabetes, chronic opioid use, psychiatric disorders, or the inability to understand pain scoring or operate the intravenous patient-controlled analgesia (IV-PCA) device.

### 2.2. Randomization and Blinding

Patients were randomized to either the ropivacaine-only group or the ropivacaine with dexamethasone group. An independent researcher, not involved in patient enrollment, intervention, or outcome assessment, generated the allocation sequence using a computer-generated randomization table. To ensure allocation concealment, group assignments were placed in sequentially numbered, sealed, opaque envelopes, which were opened only immediately before the intervention by an anesthesiologist who was not involved in the data collection or analysis.

Blinding was maintained for patients, the anesthesiologist administering the RSB, the surgeon, and the resident responsible for postoperative outcome assessment. Study medications were prepared in identical syringes by a separate anesthesiologist to ensure that both the anesthesiologist performing the block and the patients remained blinded to the group allocation.

### 2.3. Anesthesia

General anesthesia was induced with glycopyrrolate (0.2 mg), propofol (2 mg/kg), fentanyl (1 μg/kg), and rocuronium (0.6 mg/kg), followed by tracheal intubation. This combination was selected to achieve adequate hypnosis, analgesia, muscle relaxation, and hemodynamic stability. Glycopyrrolate (0.2 mg) was administered to reduce airway secretions and prevent vagally mediated bradycardia during laryngoscopy and intubation. Propofol (2 mg/kg) was used as the primary induction agent for its rapid onset and favorable induction profile. Fentanyl (1 μg/kg) was administered to attenuate the sympathetic response to laryngoscopy and intubation. Rocuronium (0.6 mg/kg) was given to provide optimal intubating conditions within 60 to 90 s [28]. Anesthesia was maintained with 50% oxygen in air and sevoflurane, with the bispectral index maintained between 40 and 60. Additional fentanyl boluses were administered as needed, up to a maximum of 2 μg/kg. Continuous remifentanil infusion was avoided due to its association with postoperative hyperalgesia [29]. All operations were performed by a single surgeon. At the conclusion of surgery, 200 mg intravenous sugammadex was administered to reverse neuromuscular blockade. Sugammadex effectively reverses moderate to deep neuromuscular blockade induced by rocuronium at doses of 2–4 mg/kg, depending on the depth of blockade [28]. Additionally, 0.3 mg intravenous ramosetron, a 5-HT_3_ receptor antagonist, was administered for prophylaxis against PONV [28]. Tracheal extubation was performed in the operating room after confirming full neuromuscular recovery.

### 2.4. Rectus Sheath Block Technique

All RSBs were performed by a single anesthesiologist in the operating room immediately after wound closure, under sterile conditions. A linear array ultrasound probe (Sonosite, Bothell, WA, USA) was placed transversely at or just above the umbilicus, with an imaging depth of 4–6 cm. A 22 G Tuohy needle was inserted a few millimeters from the probe using an in-plane technique at an approximate 45° angle to the skin. The rectus muscle and two hyperechoic lines representing the posterior rectus sheath and transversalis fascia were identified. Under direct visualization, the needle was advanced into the target plane. Here, 20 mL of 0.25% ropivacaine, with or without 4 mg dexamethasone according to the group assignment, was injected to hydrodissect the rectus muscle from the posterior sheath. The procedure was then repeated on the contralateral side. Block failure was determined in the post-anesthesia care unit (PACU) if cold sensation was not diminished in the abdominal area [30].

### 2.5. Postoperative Pain Management

Pain was assessed using a numeric rating scale (NRS) ranging from 0 (no pain) to 10 (worst imaginable pain). The well-validated visual analogue scale and the NRS demonstrate good agreement and equivalent sensitivity for acute postoperative pain assessment, both outperforming the four-point verbal categorical rating scale [31]. An IV-PCA device (Accumate^®^ 1200; Woo Young Meditech, Seoul, Republic of Korea) was initiated in the PACU, delivering a continuous infusion of 1 mL/h and a 1 mL bolus (100 mL solution containing 20 µg/kg fentanyl and 0.3 mg ramosetron), with a 15 min lockout interval. Patients were instructed to activate the PCA when the RSB no longer provided adequate analgesia. PCA usage data were collected using AccuLinker software (Accumate^®^ 1200 version 1.1; Woo Young Meditech, Seoul, Republic of Korea).

### 2.6. Outcome Assessment

Primary outcomes were total fentanyl dose administered via IV-PCA and the NRS pain scores at 3, 6, 12, 18, 24, and 48 h postoperatively. A resident assessed pain scores and monitored for complications at each time point. The secondary outcome was the incidence of rebound pain, defined as a transition from well-controlled to severe pain, typically occurring within 12–24 h after nerve block resolution. Specifically, rebound pain incidence was determined by identifying patients whose pain increased from mild (NRS ≤ 3) at the last effective block assessment (3 h postoperatively) to severe (NRS ≥ 7) within 24 h following the PNB [11]. Additionally, the incidence of PONV within 24 h and any RSB-related adverse events were recorded.

### 2.7. Statistical Analysis

An a priori power analysis was conducted using G*Power, version 3.1 (Heinrich Heine University, Düsseldorf, Germany). Based on a previous study reporting a mean pain score of 3.54 ± 0.98 at 6 h postoperatively in patients receiving a single-injection RSB [32], and assuming a 30% pain reduction with dexamethasone, a sample size of 23 patients per group was required to achieve 95% power at a two-tailed α of 0.05. Allowing for a 10% dropout rate, 26 patients per group (*n* = 52) were enrolled.

Statistical analyses were conducted using SPSS version 18.0 (IBM Corp., Chicago, IL, USA). The Shapiro–Wilk test was used to assess the normality of continuous variables. Continuous data are presented as the mean ± standard deviation or median with interquartile range (IQR), as appropriate. Categorical variables are reported as frequencies and percentages. Group comparisons were performed using the Student’s t-test or Mann–Whitney U test for continuous variables and the chi-squared test for categorical variables. A *p*-value < 0.05 was considered statistically significant. Missing data were handled with a complete-case analysis. The number and reasons for missing data were recorded, and sensitivity analyses were conducted to assess their impact.

## 3. Results

**Figure 1:**
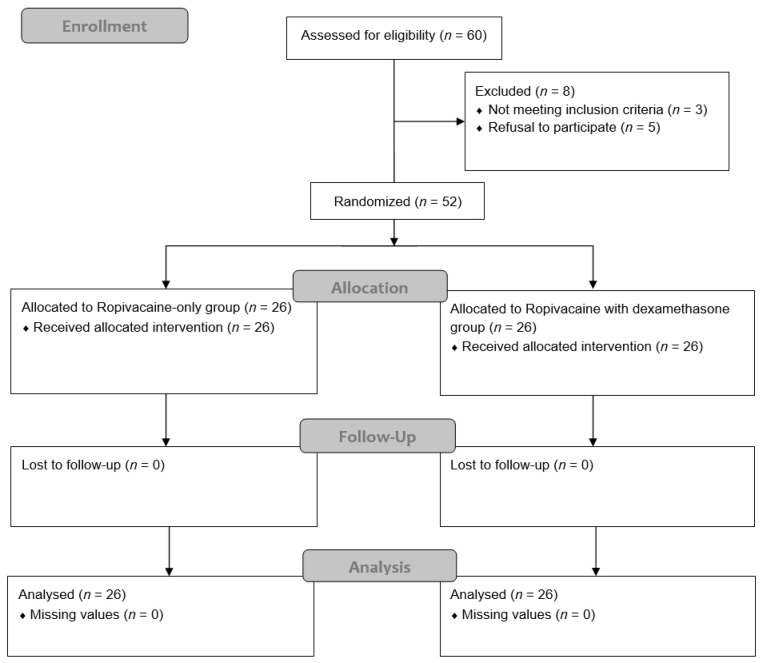
CONSORT flow diagram.

A total of 60 patients scheduled for radical cystectomy were screened for eligibility. Of these, three did not meet the inclusion criteria and five declined participation. Thus, 52 patients were enrolled, with 26 being assigned to the ropivacaine-only group and 26 to the ropivacaine with dexamethasone group. No patients were lost to follow-up or had missing data during the 48 h observation period (Figure 1).

Baseline demographic and clinical characteristics were similar between groups, with no statistically significant differences (Table 1).

### 3.1. Primary Outcomes

Total fentanyl consumption via IV-PCA was significantly lower in the ropivacaine with dexamethasone group at all time points except 3 and 6 h postoperatively. Median pain scores were also consistently lower in the ropivacaine with dexamethasone group, except at the 3 and 18 h assessments (Table 2).

### 3.2. Secondary Outcomes

Rebound pain occurred in eight patients (30.8%) in the ropivacaine-only group and in one patient (3.8%) in the ropivacaine with dexamethasone group, representing a statistically significant difference.

As is shown in Table 3, there were no significant differences in adverse events between the two groups, including PONV and nerve block-related complications.

## 4. Discussion

This study found that adding dexamethasone to an RSB significantly reduced postoperative fentanyl consumption and decreased pain scores at most time points, except at 3 h postoperatively. The incidence of rebound pain was also significantly lower in the ropivacaine with dexamethasone group compared to the ropivacaine-only group.

RSBs have become increasingly popular with the adoption of ultrasound-guided techniques for procedures involving midline abdominal incisions. An RSB is commonly performed with general anesthesia to decrease opioid requirements and improve postoperative pain control [27,33]. Although RSB efficacy has varied, several studies have demonstrated analgesic benefits in laparotomy. For instance, in patients undergoing open gastrectomy, an RSB reduced intraoperative remifentanil use and decreased PCA bolus demand during the first 2 h postoperatively [34]. Similarly, in laparotomies for mesenteric vascular occlusion, an RSB significantly lowered pain scores at 2, 4, and 6 h postoperatively and reduced opioid requirements over 24 h [35]. Bashandy et al. [36] reported significantly lower PACU pain scores and reduced morphine use over the first two postoperative days in patients receiving an RSB versus general anesthesia alone for midline procedures. However, most previous studies have shown that the analgesic effects of an RSB are most prominent within the first 6 h postoperatively, with the opioid-sparing benefits lasting up to 24 h. In contrast, our study is the first to examine the addition of perineural dexamethasone as an adjuvant to an RSB. Our results showed reductions in both pain scores and opioid consumption for up to 48 h, along with a significantly lower incidence of rebound pain. Unlike an RSB, several studies have evaluated dexamethasone as an adjunct to the TAP block, another widely used fascial plane block. A meta-analysis found modest reductions in pain scores at 2, 6, and 12 h postoperatively and decreased 24 h opioid use with perineural dexamethasone [37]. These findings support the analgesic-enhancing and duration-prolonging effects of dexamethasone in fascial plane blocks, consistent with our results.

In our study, fentanyl consumption and pain scores at 3 h were similar between groups, likely reflecting the initial analgesic effect of the local anesthetic in both groups. This observation aligns with a meta-analysis reporting that dexamethasone reduced pain at rest during the intermediate (8–12 h) and late (24 h) postoperative periods and decreased morphine requirements [38]. Additionally, the use of a higher concentration and volume of local anesthetic for single-injection PNBs may yield greater analgesic efficacy during the immediate postoperative period, resulting in enhanced opioid-sparing effects early after surgery [24]. Current recommendations for ultrasound-guided RSBs suggest volumes of 0.1–0.2 mL/kg (typically 15–20 mL per side in adults), with the concentration adjusted to avoid toxicity [39]. In our study, a relatively high volume of local anesthetic (20 mL of 0.25% ropivacaine per side) was administered. Consequently, an RSB with local anesthetic alone provided effective pain relief during the immediate postoperative period following radical cystectomy.

Preoperative RSBs have been associated with a delayed time to first analgesic request and reduced analgesic requirements within 24 h in laparoscopic surgery [40]. However, in this study, the RSB was performed after skin closure and before extubation. This approach was based on growing evidence that preemptive analgesia provides limited benefit, despite its theoretical rationale for preventing central sensitization. Recent studies have highlighted that the duration and continuity of analgesia are more important determinants of pain outcomes than the timing of initiation [41,42]. Animal models of incisional pain have consistently demonstrated that a single analgesic intervention—whether peripheral or neuraxial—does not reduce postoperative pain beyond its pharmacologic duration [43]. Clinical trials have reported similar findings, and a systematic review by Møiniche et al. also concluded that preemptive analgesia does not provide significant benefits for postoperative pain control [41]. Once nociceptive afferent blockade resolves, central sensitization can recur [41]. Therefore, ensuring an adequate duration and efficacy of analgesia throughout the perioperative period is essential. Additionally, given the prolonged duration of radical cystectomy, initiating an RSB at the beginning of surgery could lead to rebound pain during the immediate postoperative period, potentially compromising pain control. Performing an RSB at the end of surgery may therefore optimize the timing and effectiveness of analgesia.

Preventive analgesia encompasses perioperative interventions that mitigate pain-induced sensitization [44]. Central neuronal sensitization contributes substantially to postoperative pain and may play a role in persistent post-surgical pain [45]. Continuous PNBs have been proposed to prevent rebound pain by providing prolonged afferent blockade and reducing the risk of central sensitization [30]. However, continuous nerve catheters are more time consuming, technically challenging, and costly, and they carry risks such as displacement [46,47]. In contrast, single-injection PNBs are easier to administer, provide reliable and immediate analgesia, and reduce opioid requirements, despite their shorter duration [48].

A meta-analysis demonstrated that both intravenous and perineural dexamethasone reduce rebound pain after PNBs, with perineural administration being more effective [49]. Additional meta-analyses have confirmed that perineural dexamethasone prolongs block duration more than intravenous administration [49,50,51]. Proposed mechanisms include decreased systemic absorption of local anesthetic due to local vasoconstriction [52], inhibition of neuronal potassium channels [53], and suppression of nociceptive C-fiber activity [54]. In a mouse sciatic nerve block model, perineural dexamethasone also reduced bupivacaine-induced rebound hyperalgesia and neurotoxicity by preserving Schwann cell and myelin integrity [55]. Clinically, rebound pain presents a significant challenge because it may diminish the initial analgesic benefits of regional anesthesia. Patients frequently experience a sudden increase in pain within 12 to 24 h after block resolution, leading to distress and increased reliance on systemic analgesics, including opioids [56]. The severity and timing of rebound pain are therefore critical considerations for postoperative pain management protocols, as inadequate anticipation or management can negatively impact patient satisfaction and recovery. Furthermore, rebound pain’s consequences extend beyond immediate discomfort, potentially contributing to heightened pain sensitivity and delays in mobilization and rehabilitation during postoperative recovery [57].

The analgesic efficacy of dexamethasone increases with the dose until reaching a plateau. One meta-analysis found that 4 mg of perineural dexamethasone extended analgesia from 11.1 to 16.5 h, while 8 mg provided only one additional hour [58]. Based on these results, we selected a 4 mg dose of dexamethasone for this study.

Several studies have compared the analgesic efficacy of dexamethasone and alpha-2 agonists, particularly dexmedetomidine, as adjuvants to local anesthetics in regional anesthesia. Although no study has directly compared these agents in radical cystectomy, evidence from other surgical settings offers valuable insight. For example, Gao et al. [59] conducted a randomized trial in patients undergoing video-assisted thoracoscopic lobectomy, comparing ropivacaine combined with dexmedetomidine or dexamethasone for an erector spinae plane block. They found that dexmedetomidine significantly prolonged the sensory block duration, improved acute pain control, reduced rescue analgesia requirements, and shortened the hospital stay compared to dexamethasone. In contrast, Singh et al. [60] reported that, in upper-limb procedures using a supraclavicular brachial plexus block, the onset times and durations of sensory and motor blockade, postoperative pain scores, duration of analgesia, and total analgesic consumption were comparable between the dexmedetomidine and dexamethasone groups. Additionally, Venkatraman et al. [61] showed that dexamethasone resulted in the longest duration of postoperative analgesia, surpassing both dexmedetomidine and morphine when combined with ropivacaine. In their study, the onset of sensory and motor blockade was faster with dexmedetomidine than dexamethasone, while the 24 h tramadol and paracetamol consumption was lower in the morphine and dexamethasone groups compared to dexmedetomidine.

The estimated incidence of PONV in surgical patients is approximately 30%, but this can exceed 80% in high-risk populations [62]. Risk factors include female sex, prior PONV or motion sickness, nonsmoking status, and a younger age. Laparoscopic, bariatric, gynecologic, and cholecystectomy procedures are also associated with an increased risk [62]. The median incidence of PONV in the PACU has been reported to be lower in RSB recipients compared to general anesthesia alone [36], and one meta-analysis reported a 72% reduction in PONV with perineural dexamethasone [37]. However, our study observed a low overall incidence of PONV, with no significant difference between the groups. This likely reflects our cohort’s lower baseline risk, as most participants were older male patients undergoing a radical cystectomy and routinely received 5-HT3 antagonists.

As previously discussed, dexamethasone has been extensively studied as an adjuvant to local anesthetics in regional anesthesia due to its anti-inflammatory, analgesic, and antiemetic effects. Multiple meta-analyses have shown that adding dexamethasone to PNBs significantly reduces postoperative pain scores and opioid consumption while prolonging analgesia duration [13,37]. Dexamethasone also effectively reduces PONV incidence, a common concern in opioid-based anesthesia, thereby improving patient satisfaction and postoperative recovery [63]. Despite these clinical benefits, dexamethasone use requires caution because of potential adverse effects, especially with repeated or high doses. These include immunosuppression, hyperglycemia, delayed wound healing, and adrenal suppression. Careful patient selection and dose adjustment are therefore essential, particularly in individuals with diabetes, active infections, or compromised immune function. Several meta-analyses have reported that, while dexamethasone administration is associated with significantly increased blood glucose levels during the first postoperative day, it does not increase the infection risk or delay wound healing [19,20]. Dexamethasone is contraindicated in patients with known corticosteroid hypersensitivity, systemic fungal infections, or untreated systemic infections. It should also be avoided or used with caution in patients with active peptic ulcer disease, uncontrolled diabetes mellitus, severe psychiatric disorders, congestive heart failure, severe hypertension, or glaucoma [64].

This study had several limitations. First, the sample size was relatively small and insufficiently powered to detect differences in all outcomes. Second, postoperative pain assessment via the NRS is inherently subjective and may be influenced by individual variability. Third, these findings are specific to an RSB and may not be generalizable to other regional anesthesia techniques or surgical procedures. Fourth, the lack of a non-block control group limits interpretation of the net benefit of an RSB in evaluating rebound pain. Fifth, intravenous dexamethasone was not assessed, so its comparative effectiveness remains uncertain. Sixth, the 48 h follow-up limited our ability to evaluate long-term outcomes such as chronic pain or prolonged opioid use. Seventh, we did not account for factors such as intraoperative anesthetic management, surgical technique variability, or psychological factors, all of which may influence postoperative pain perception and analgesic requirements. Eighth, because all procedures were performed by a single surgeon and anesthesiologist at one institution, the study’s external validity may be limited. Ninth, although there were no significant differences between groups, intraoperative opioid administration remains a potential confounder affecting postoperative pain and opioid consumption. Finally, we did not measure postoperative glucose levels, as these are influenced by multiple confounding factors such as surgical stress, anesthetic agents, and perioperative medications, although we sought to standardize these variables in this study.

## 5. Conclusions

This study demonstrated that the addition of perineural dexamethasone to an RSB significantly reduced postoperative opioid usage and pain scores in patients undergoing a radical cystectomy, with analgesic benefits lasting up to 48 h. Additionally, the incidence of rebound pain was markedly lower in the ropivacaine with dexamethasone group compared to the ropivacaine-only group. These findings suggest that perineural dexamethasone is a safe and effective adjuvant for enhancing the analgesic efficacy of an RSB in this surgical population.

Future research should directly compare perineural and intravenous dexamethasone, as well as preoperative versus postoperative RSBs, to determine the optimal analgesic strategy for radical cystectomy. Additionally, head-to-head trials comparing dexamethasone with other adjuvants such as alpha-2 agonists and NSAIDs are warranted. Long-term outcomes and multimodal combination strategies should also be explored. Given the limitations of this single-center, single-surgeon, and single-anesthesiologist study design, multicenter randomized trials are necessary to enhance the generalizability and external validity of these findings.

## Figures and Tables

**Table 1:** Demographic characteristics of study participants.

	Ropivacaine-Only Group (*n* = 26)	Ropivacaine with Dexamethasone Group (*n* = 26)	*p*-Value
Age (y)	68.50 [8.50]	67.50 [9.25]	0.275
Height (cm)	167.00 [9.65]	167.10 [8.98]	0.498
Weight (kg)	66.66 ± 11.86	65.66 ± 10.17	0.745
Sex (M/F)	21 (81)/5 (19)	21 (81)/5 (19)	1.000
Operation duration (min)	252.50 [61.25]	225.00 [31.25]	0.184
Anesthesia duration (min)	287.50 [67.50]	265.00 [33.75]	0.152
ASA physical status (1/2/3)	0 (0)/23 (88)/3 (12)	3 (11)/21 (81)/2 (8)	0.193
Diabetes mellitus	7 (27)	9 (35)	0.548
Hypertension	16 (62)	13 (50)	0.402
Pulmonary disease	1 (4)	3 (12)	0.298
Intraoperative fentanyl (μg)	100.00 [50.00]	100.00 [50.00]	0.627

Data are presented as mean ± standard deviation, median [interquartile range], or number of patients (%). ASA, American Society of Anesthesiologists.

**Table 2:** Postoperative total fentanyl use and pain scores.

	Ropivacaine-Only Group (*n* = 26)	Ropivacaine with Dexamethasone Group (*n* = 26)	*p*-Value
Total fentanyl dose (µg)			
Postoperative 3 h	101.70 [43.05]	84.44 [33.72]	0.164
Postoperative 6 h	179.20 [138.60]	148.50 [100.03]	0.059
Postoperative 12 h	294.95 [173.48]	247.95 [131.98]	**0.041**
Postoperative 18 h	420.35 [259.30]	359.80 [140.50]	**0.022**
Postoperative 24 h	521.50 [332.58]	459.80 [175.42]	**0.032**
Postoperative 48 h	913.70 [246.95]	806.30 [300.45]	**0.024**
Numeric rating scale			
Postoperative 3 h	2.00 [2.00]	2.00 [1.00]	0.113
Postoperative 6 h	6.00 [2.25]	4.50 [2.25]	**0.002**
Postoperative 12 h	5.00 [2.00]	3.00 [2.00]	**0.000**
Postoperative 18 h	4.00 [2.00]	3.00 [2.00]	0.057
Postoperative 24 h	3.00 [1.50]	2.50 [1.25]	**0.024**
Postoperative 48 h	2.00 [2.25]	1.00 [1.00]	**0.003**

Data are presented as median [interquartile range]. Boldface values indicate statistical significance.

**Table 3:** Adverse events.

	Ropivacaine-Only Group (*n* = 26)	Ropivacaine with Dexamethasone (*n* = 26)	*p*-Value
Postoperative nausea and vomiting	3 (12)	2 (8)	0.638
Wound infection	0 (0)	0 (0)	1.000
Local anesthetic systemic toxicity	0 (0)	0 (0)	1.000
Perineural infection or abscess	0 (0)	0 (0)	1.000
Pruritus	0 (0)	0 (0)	1.000

Data are presented as number of patients (%).

## Data Availability

Data available on request due to restrictions.

## Abbreviations

The following abbreviations are used in this manuscript:

NSAIDs	Nonsteroidal anti-inflammatory drugs
PNB	Peripheral nerve block
PONV	Postoperative nausea and vomiting
RSB	Rectus sheath block
TAP	Transversus abdominis plane
PACU	Post-anesthesia care unit
NRS	Numeric rating scale
IV-PCA	Intravenous patient-controlled analgesia
